# The moderating role of physical fitness in the relationship between sugar-sweetened beverage consumption and adiposity in schoolchildren

**DOI:** 10.1038/s41598-022-23092-1

**Published:** 2022-11-03

**Authors:** José Francisco López-Gil, Iván Cavero-Redondo, Mairena Sánchez-López, José Alberto Martínez-Hortelano, Carlos Berlanga-Macias, Alba Soriano-Cano, Vicente Martínez-Vizcaíno

**Affiliations:** 1 Health and Social Research Center, Universidad de Castilla-La Mancha, Cuenca, Spain; 2 Facultad de Ciencias de La Salud, Universidad Autónoma de Chile, Talca, Chile; 3 Facultad de Enfermería, Universidad de Alcalá de Henares, Alcalá de Henares, Spain

**Keywords:** Nutrition, Paediatrics, Weight management

## Abstract

The mediating and moderating associations of cardiorespiratory fitness (CRF) and handgrip strength on the association between dietary patterns and several health outcomes have been previously studied. For instance, handgrip strength has been found as a moderator of the relationship between excess weight and cardiometabolic risk factors in young adults. Similarly, CRF has been shown as a mediator of the association between diet and obesity in children. However, to our knowledge, the role of CRF and handgrip strength on the association between sugar-sweetened beverage (SSB) consumption and adiposity is still unclear. The aim of this study was to determine whether CRF and handgrip strength moderate the association between SSB consumption and adiposity in a population-based sample of Spanish schoolchildren. This cross-sectional study involved 475 schoolchildren (52.0% girls), aged 8–12, from ten schools in Cuenca (Spain). Adiposity was determined as body fat (in kg), which was measured using a bioimpedance analysis system. Data on SSB consumption were gathered by using the Children’s Eating Habits Questionnaire, which was completed by parents. The CRF level was determined by the 20-m Shuttle Run test and Nevill’s curvilinear allometric model. Handgrip strength was determined using a digital dynamometer with adjustable grip. For each unit (in ml/kg/min) of CRF increased, the association between SSB consumption and adiposity was moderated (B = − 0.09, CI 95% − 0.14 to − 0.04). This significant moderation was also found for each 0.01 unit of increased normalized handgrip strength (B = − 0.07; CI 95% − 0.11 to − 0.02). Similarly, the Johnson-Neymann technique established three different regions. The first region shows that the association of SSB consumption on adiposity in participants who had levels of CRF < 43.4 ml/kg/min or handgrip strength < 0.34 was greater and statistically significant. The second region (43.4–57.4 ml/kg/min for CRF; 0.34–0.58 for normalized handgrip strength) depicted that the association between SSB consumption and adiposity was not statistically significant in those with a CRF level or normalized handgrip strength between the lower and upper thresholds. The third region was found at > 57.4 ml/kg/min (for CRF level) and > 0.58 (for normalized handgrip strength), indicating that the association between SSB consumption and adiposity was lower and statistically significant in children above these moderator values. Our results showed that certain levels of CRF and normalized handgrip strength moderate the association between SSB consumption and adiposity in a sample of Spanish schoolchildren. It might be possible that higher physical fitness level in childhood may contribute to reducing the association between SSB consumption and adiposity.

## Introduction

The detrimental effects of free sugar consumption on health have aroused enthusiastic interest in recent years^1^, such that the World Health Organization (WHO) is calling for worldwide action to reduce the consumption of sugar-sweetened beverages (SSBs)^2^. In this respect, the WHO recommends that the consumption of free sugars should not exceed 10% of total daily energy intake^3^ because the consumption of SSBs, candies and sweet snacks may increase the risk of weight gain^4^ and insulin resistance^5^. Among the environmental factors recognized as influencing excess weight in children, the availability of SSBs has been identified as one of the most influential^6^. In Spain, SSBs and soft drinks are consumed by 81% of the children weekly^7^.

Cardiorespiratory fitness (CRF)^8^ and muscular strength^9^ have important implications for health in young populations and later in life. A recent meta-analysis found a moderate inverse relationship between CRF and adiposity later in life^10^. Similarly, another meta-analysis reported a moderate inverse association between muscular fitness and certain anthropometric indicators (e.g., skinfold thickness) over the years^9^. Previous studies among Spanish children have reported the proportion that meeting with the criterion-referenced standards for physical fitness^11,12^. For instance, Castro-Piñero et al.^13^ found in their study including children aged 6–10 years that 37% of boys and 70% of girls met the criterion-referenced standards for CRF (i.e., 42 and 35 ml/kg/min cut-off points in boys and girls, respectively). Similarly, Valenzuela et al.^12^ found in their study that the 60% of the European children analyzed (including Spanish children) met these same CRF criterion-referenced standards.

It has been suggested that a balanced diet (e.g., low in SSBs) would have major physical fitness benefits during childhood and adolescence^14^. Supporting this notion, CRF has been found to be related to lower SSB consumption in adolescents (only statistically significant in girls)^15^. Moreover, higher consumption of SSBs was significantly associated with lower handgrip strength (in middle-aged and older adults)^16^.

The mediating and moderating associations of CRF and handgrip strength on the association between dietary patterns and several health outcomes have been previously studied^17–20^. However, to our knowledge, the role of CRF and handgrip strength on the association between SSB consumption and adiposity is still unclear. Therefore, it could be suggested that higher levels of CRF and muscle strength could help to partially counteract the association of SSB consumption on adiposity. Thus, the aim of this study was to determine whether CRF and normalized handgrip strength moderate the association between adiposity and SSB consumption in a population-based sample of Spanish schoolchildren.

## Material and methods

### Design and participants

This study involved 475 schoolchildren (52.0% girls), aged 8–12 years, from ten schools in Cuenca (Spain). All fourth- and fifth-grade children in these schools were invited to participate. Exclusion criteria were applied as follows: (a) children with Spanish learning difficulties; (b) children with severe mental or physical disorders; and (c) children with chronic disorders (e.g., heart disease, asthma, diabetes). The present study was approved by The Clinical Research Ethics Committee of the Virgen de la Luz Hospital in Cuenca (REG: 2016/PI021) and performed in accordance with the Declaration of Helsinki. An informed consent was obtained from parents of all participants involved in the study. Parents were informed that they could withdraw their agreement to participate at any time. Moreover, schoolchildren were asked (verbally) to give consent before performing each test.

### Procedures

#### Anthropometric data

Anthropometric variables were determined twice, and their averages were used for subsequent analyses. For height measurements, a stadiometer (Seca 222) was used with children barefoot and in an upright position while the sagittal midline touched the backboard. Children were encouraged to look straight ahead, and the line of sight was parallel to the ground. Weight was computed with a scale (Seca 861) with the child barefoot and in light clothing. Body mass index (BMI) was determined by dividing weight (kg) and height (m^2^).

#### Sugar-sweetened beverages consumption (exposure)

SSB consumption (i.e., soft drinks) was measured using the Children’s Eating Habits Questionnaire (CEHQ)^21^. The CEHQ has demonstrated to provide acceptable reproducibility of food estimates at group level^21^. This questionnaire was completed by parents, who had the following six response options: never; < 1 time per week; 1 time per week; 2–4 times per week; 5–6 times per week; 1 time per day; and > 1 time per day. For further analyses, we collapsed these categories into: (a) never; (b) sometimes (“1 time per week”, “1 time per week”, “2–4 times per week”, and “5–6 times per week”), and (c) daily (“1 time per day”, and “ > 1 time per day”).

#### Body composition (outcome)

Adiposity was determined as body fat (BF) (in kg), which was measured using an 8-electrode Tanita Segmental-418 bioimpedance analysis system (Tanita Corp., Tokyo, Japan)^22^, under controlled humidity and temperature settings. Furthermore, measurements were performed after fasting, after urinating, and after a 15-min rest period.

#### Physical fitness (moderator variable)

CRF was determined by the 20-m Shuttle Run test. For this test, two lines were drawn 20 m apart on the ground. The 20-m Shuttle Run test is an acceptable, feasible, and scalable measure of CRF and functional/exercise capacity, and has moderate criterion validity and high to very high reliability^23^. Children were encouraged to run backwards and forwards following the sound signal of a pre-recorded tape. This sound started at a cadence of 6.5 km/h and increased by 0.5 km/h each minute. The test was finished when children were not able to reach the lines before the signal sounded twice. On the basis of these data, maximal oxygen intake (VO_2_max) was calculated using the curvilinear allometric model by Nevill et al.^24^. Because of the high variability in body composition of children, we used this model instead of the Léger et al. formula^25^ to improve the fit and validity of the 20-m Shuttle Run Test as a predictor of CRF in youth^24^.

Furthermore, the handgrip strength was determined in kilograms using the digital dynamometer with adjustable grip TKK 5401 Grip- DW (Takeya, Tokyo, Japan). The handgrip strength test has high-to-very high construct validity with total muscle strength in healthy children (among other parameters)^26^. The test was performed twice with the left hand and twice with the right hand; the average of the four measurements was computed. Handgrip data were normalized by dividing absolute handgrip strength (kg) by body weight (kg).

To categorize physical fitness, sex- and age-specific European normative values for physical fitness in children and adolescents^27^ were applied. Thus, participants with 20-m Shuttle Run Test (stages) or absolute handgrip strength (kg) with values at or above the 60th percentile were considered "high CRF" or “high handgrip strength”, respectively.

#### Covariates

Age and sex were self-reported by the participants. Socioeconomic status (SES) was estimated by the Spanish Epidemiology Society Scale^28^. Thus, mothers and fathers reported their respective educational levels and employment status, and an index considering both was calculated^28^. Somatic maturity was estimated by following the prediction models by Moore et al.^29^. As adiposity, fat-free mass was determined by a bioimpedance analysis system^22^. Furthermore, additional food groups (e.g., fruits, vegetables, sweets) assessed by the above-mentioned CEHQ^21^, were also considered as a covariates.

### Statistical analyses

Descriptive data are expressed as number and percentage for categorical variables, and as mean and standard deviation (SD) for continuous variables. Moderation analyses were conducted using PROCESS macro 4.0 in SPSS (IBM SPSS Statistics for Windows, Version 25.0, Armonk, NY, USA). The PROCESS macro applies ordinary least squares (OLS) analysis to estimate moderation models (model 1 in PROCESS) using BF (in kg) as the dependent variable (*Y*), SSBs as the independent variable (*X*), and CRF and normalized handgrip strength as moderator variables (*W*) (method for probing moderation mean + /− 1 SD), with a bootstrapping resampling approach with 10,000 samples (to estimate standard errors and confidence intervals)^30^. The theorical model is represented as follows: *Y* = *iY* + *b*_1_*X* + *b*_2_*W* + *b*_3_*XW* + *eY*, where *b*_1_, *b*_2_, and *b*_3_ are estimated regression coefficients, *eY* is an estimation error, and *iY* is a regression intercept^31^. This model estimates *Y* (outcome) from two priors *X* (exposure) and *W* (moderator variable). This equation was applied for CRF and normalized handgrip strength, independently. In addition, the Johnson-Neyman (J-N) procedure was performed to check interactions and to find regions of statistical significance and different point estimates of the moderators with an alpha setting at 0.05 for the J-N procedure and confidence intervals^32^. All analyses were adjusted for sex, age, SES, somatic maturity, fruit consumption, vegetables consumption, and sweets consumption. Similarly, analyses were weighted to consider the population size of the different clusters (schools).

### Ethics declarations

The Clinical Research Ethics Committee of the Virgen de la Luz Hospital in Cuenca approved the present study (REG: 2016/PI021). Parents were informed that they could withdraw their agreement to participate at any time.

### Informed consent

Informed consent was obtained from parents/legal guardians of all participants involved in the study.

## Results

Table 1 shows the characteristics of the study participants. The mean BF (in kg) was 24.0 ± 6.7. Twenty-five percent of the sample stated that they had never consumed SSBs. For physical fitness, the mean CRF and normalized handgrip strength were 7.9 ± 1.6 ml/kg/min and 0.35 ± 0.10, respectively. The proportion of participants with high CRF and high handgrip strength was 31.8% and 9.3%, respectively.

**Table 1:** Characteristics of the study participants (N = 475).

Variable	M ± SD/n (%)	CI 95%
Age (years)	9.6 ± 0.7	9.5–9.7
**Sex**		
Girls	247 (52.0)	47.1–55.9
Boys	228 (48.0)	44.1–52.9
**Anthropometric data**		
Weight (kg)	36.5 ± 9.8)	35.63–37.35
Height (cm)	140.8 ± 7.2)	140.2–141.4
**Body composition**		
BF (kg)	9.3 ± 5.3	23.4–24.6
FFM (kg)	27.2 ± 5.0	26.8–27.7
**SSB consumption**		
Never	124 (26.1)	21.9–30.0
Sometimes	299 (62.9)	21.5–29.2
Daily	52 (10.9)	16.6–23.6
**Physical fitness**		
20-m Shuttle Run Test	3.7 ± 1.8	3.6–4.0
CRF (ml/kg/min)^a^	44.6 ± 8.3	43.9–45.5
High CRF^b^	136 (31.8)	27.7–36.0
Handgrip strength (kg)	12.3 ± 3.5	12.00–12.62
Handgrip strength (kg)/Body mass (kg)	0.35 ± 0.10	0.34–0.36
High handgrip strength^b^	46 (9.3)	6.8–11.9

*BF* body fat, *BMI* Body mass index, *CI* confident interval, *CRF* cardiorespiratory fitness, *FFM* fat-free mass, *M* mean, *SD* standard deviation, *SSB* Sugar-sweetened beverage.

^a^According to the curvilineal allometric model by Nevill et al.^24^.

^b^According to the European normative values by Tomkinson et al.^27^.

**Figure 1:**
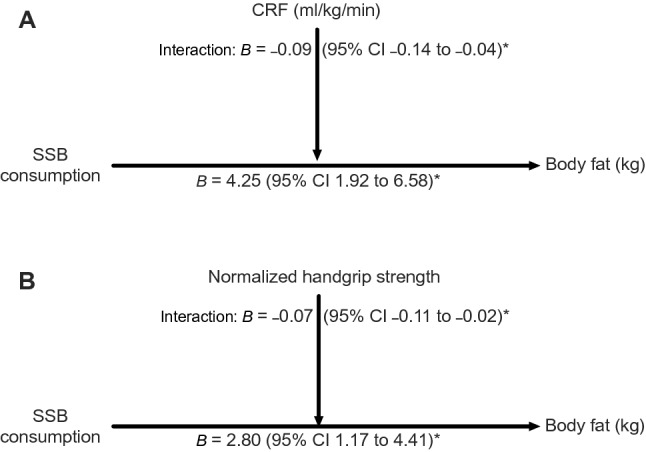
Moderation analyses in which cardiorespiratory fitness and normalized handgrip strength moderate the association between sugar-sweetened beverage consumption and adiposity. Beta presented as unstandardized regression coefficients and 95% confidence interval. Adjusted for sex, age, socioeconomic status, somatic maturity, fat-free mass, fruit consumption, vegetables consumption, and sweets consumption. *p < 0.05.

Figure 1 depicts the moderation analysis following the OLS regression. The unstandardized beta coefficients represent the amount by which the outcome changes if we change the exposure by one unit (i.e., for each category of higher SSB consumption) while keeping the other independent variables constant (i.e., the moderator variable and the covariates included in the model). This finding indicated a significant association between SSB consumption and adiposity in Spanish schoolchildren. Likewise, for each unit (in ml/kg/min) of CRF increased, the association between SSB consumption and adiposity was moderated (*B* = –0.09, CI 95% –0.14 to –0.04) (Fig. 1A). This significant moderation was also found for each 0.01 unit of normalized handgrip strength increased (*B* = –0.07; CI 95% –0.11 to –0.02) (Fig. 1B). To aid interpretation, the association between SSB consumption (i.e., never, sometimes, or daily) and adiposity, according to the established CRF or handgrip categories (i.e., values at –1 SD, mean, or + 1 SD established by the pick-a-point approach) is found in Figure S1.

**Figure 2:**
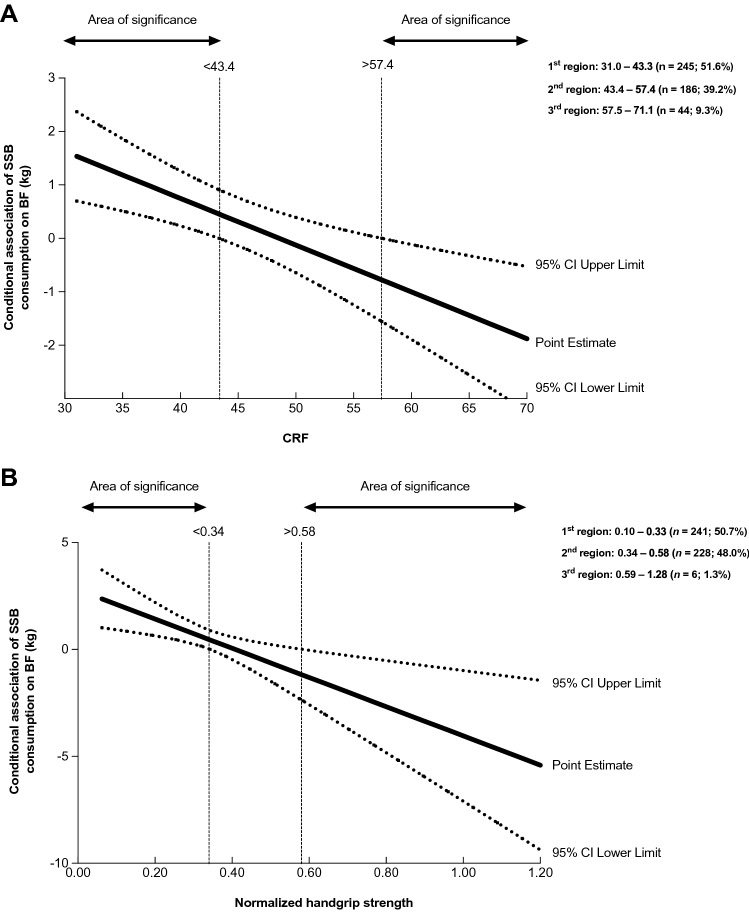
Moderating role of cardiorespiratory fitness and normalized handgrip strength on the association between sugar-sweetened beverage consumption and adiposity using the Johnson-Neyman procedure. Data are showed as a regression point estimate (beta unstandardized) and 95% confidence intervals. Adjusted for sex, age, socioeconomic status, somatic maturity, fat-free mass, fruit consumption, vegetables consumption, and sweets consumption.

Finally, the moderating role of CRF and normalized handgrip strength in the association between SSB consumption and BF (in kg) is shown in Fig. 2. The Johnson-Neyman procedure indicated different point estimates of CRF as a moderator, using the slope and the different significant regions (Fig. 2A). First, the region exposed to a CRF level < 43.4 ml/kg/min indicates that the association of SSB consumption on adiposity is greater and statistically significant for schoolchildren in this region. Second, the region between 43.4 and 57.4 ml/kg/min depicts that the association was not statistically significant in those with a CRF level between the lower and upper thresholds. Third, another region found at > 57.4 ml/kg/min, which showed and opposite direction to that found in the first region (i.e., at < 43.4 ml/kg/min), denoting that the association between SSB consumption and adiposity was lower and statistically significant. Similarly, Fig. 2B describes three different moderating values for normalized handgrip strength. The first region is shown at < 0.34, indicating that the association between SSB consumption and adiposity was greater and statistically significant for those in this region. The second region was detected between 0.34 and 0.58, which revealed that the association was not statistically significant for participants with a normalized handgrip strength within this region. The third region was found at > 0.58, where an opposite direction to that found in the first region (i.e., < 0.34) was observed, indicating that the association between SSB consumption and adiposity was lower and statistically significant in schoolchildren above this estimate point.

## Discussion

The present study explored the moderating role of CRF and normalized handgrip strength on the association of SSB consumption on adiposity in Spanish schoolchildren. The main finding of this study is that the association of SSB consumption and adiposity was moderated by CRF and normalized handgrip strength. For each unit increase in these physical fitness components (i.e., CRF or normalized handgrip strength) the association between SSB consumption and adiposity was lower. In addition, two moderation cut-off points were determined for CRF: below 43.4 (higher association) and above 57.4 (lower association). Similarly, for normalized handgrip strength, two moderation cut-off points were determined: below 0.34 (higher association) and above 0.58 (lower association).

Some previous studies agree with our results. For instance, SSB consumption was associated with excess weight in a sample of Brazilian children and adolescents^33^. A longitudinal study among non-Hispanic whites in the US (girls only) showed that higher consumption of SSBs was related to higher adiposity, weight status and waist circumference^34^. In Denmark, a longitudinal study showed that the consumption of liquid sucrose was associated with changes in waist circumference and body mass index (z-score)^35^. Conversely, in this study, no interactions were found between liquid sucrose consumption and physical fitness according to obesity or body fat distribution. There are some possible reasons explaining this mismatch. For instance, body fat was assessed by BMI and waist circumference instead of bioimpedance analysis system. Both BMI and waist circumference are not specific measures of adiposity (e.g., BMI does not distinguish between muscle mass and body fat^36^). In addition, a different method to assess the CRF level (electronically braked bicycle ergometer) was used. Although electronically braked bicycle ergometer could provide more accurate estimates of CRF, these authors used different statistical methods to test the interaction between liquid sucrose and physical fitness in relation to BMI/waist circumference (i.e., linear regression analyses versus Johnson-Neyman technique). The Johnson-Neyman technique provides “areas of non-significance” in which the difference between groups is not significant and, at the same time, it allows us to identify the zones in which these differences are significant^37^.

Although some studies have shown an association between adiposity and SSB consumption or physical fitness, this is the first study to assess the moderating role of physical fitness (CRF and normalized handgrip strength) in the association between SSB consumption and adiposity. In this regard, although the specific mechanisms through which SSBs are associated with childhood obesity are not completely understood^5^, we propose three different biological mechanisms that could explain this finding.

First, it has been reported that beverages could produce less satiety than that of solid foods^38,39^. Low satiety responsiveness is one of the reasons why genetic tendency leads to increased weight gain in an obesogenic environment (i.e., an environment with high food availability)^40^. In the same line, increased physical fitness is related to high-intensity physical activity^41^ which, in turn, is a powerful component affecting the maintenance of energy balance, accounting for about 25% of total energy expenditure^42^. Supporting this notion, daily moderate-to-vigorous PA (which is closely related to a higher physical fitness^43^) appears to moderate some of the association between unhealthy diet^44^ or eating habits (e.g., breakfast^45^) on weight and fat mass. We hypothesized that children who are more physically active (and possibly more physically fit) may burn the excess calories from consumption of SSBs (among other foods).

Second, supporting the abovementioned mechanism, liquid foods do not trigger the physiological mechanisms that induce satiety, providing inaccurate and partial compensation for energy consumption in liquid form^46^. Regarding physical fitness, there is evidence that physical exercise is related to changes in intake-related peptides (e.g., ghrelin), which, in turn, will affect the drive to eat through altered perceived hunger (a conscious feeling leading to a mental impulse to eat) and modifications in postprandial satiety through an interaction with food composition^47^. Based on the above, one possible reason justifying our results is that, although children consume SSBs, higher levels of exercise act on certain intake-related peptides (e.g., ghrelin), which could be directly or indirectly related to lower adiposity.

Third, although the evidence is scarce, SSB consumption and subsequent weight changes and the development of obesity could be mediated by increased insulin release^35^. Furthermore, added liquid sugars are a risk factor of insulin resistance and impaired glucose homeostasis among young people at risk for obesity^48^. Concerning physical fitness, lower CRF levels in youth with obesity are associated with significantly greater insulin indices (insulin release response during the oral glucose tolerance test and the homeostasis model assessment of insulin resistance (HOMA-IR))^49^. Moreover, a study by Fraser et al.^50^ showed that muscular fitness and CRF during childhood predict fasting insulin levels, beta cell function, and insulin resistance in adulthood^50^. For these reasons, it is possible to speculate that, despite SSB consumption, children who have higher levels of physical fitness (e.g., CRF, handgrip strength) also have higher insulin sensitivity, which could be related to lower adiposity.

This study has several limitations that should be mentioned. First, there is not follow-up in this cohort. Second, because of the cross-sectional design of this study (which is a limitation), we are not able to assess the temporality of the exposure and outcome. Another limitation is that we did not use gas-analyzed peak oxygen uptake to evaluate CRF levels, although it is considered the physiological criterion for measuring CRF levels in children. Nonetheless, recommendations advise the use of the 20-m Shuttle Run Test to evaluate CRF levels as an international population health surveillance measure to provide more information on the health status of the youth population^23^. In addition, the frequency of foods reported by parents may be limited to times when children are at home or with their parents or primary caregivers, so children may have higher intakes of some foods in the absence of parents. Moreover, there could be information biases in relation to SSB consumption. In this regard, adults with obesity tend to underestimate carbohydrate-rich foods in particular^51^. It could be suggested that parents with obesity may underestimate their children's SSB consumption. Conversely, the main strength of this study is that, to date, it is the first study to analyze the moderating role of physical fitness (CRF and normalized handgrip strength) in the association between SSB consumption and adiposity among schoolchildren. This finding could be useful for future intervention programs or other studies with different design (e.g., longitudinal, intervention).

In conclusion, our results show that certain levels of CRF and normalized handgrip strength moderate the association between SSB consumption and adiposity in a population-based sample of Spanish schoolchildren. This finding is clinically significant since SSB consumption is one of the most frequent unhealthy patterns associated with adiposity in children. Given these findings and the low proportion of children found above the established cut-off points for both CRF and normalized handgrip strength, it might be possible to underline the significance of improving physical fitness in childhood to help to reduce the association between SSB consumption and adiposity. Notwithstanding, further studies with different designs are needed to verify cause-effect associations.

## Supplementary Information

Supplementary Legends.

Supplementary Figure S1.

## Data Availability

The data presented in this study are available on request from the corresponding author. The data are not publicly available because they belong to minors.
