# Supplementary material for: The moderating role of physical fitness in the relationship between sugar-sweetened beverage consumption and adiposity in schoolchildren
Source: Sci Rep. 2022 Nov 3;12:18630. doi: 10.1038/s41598-022-23092-1 (PMC9633592; doi:10.1038/s41598-022-23092-1)
Supplement: Supplementary file 1 — Supplementary Legends. [file 41598_2022_23092_MOESM1_ESM.docx]

**Figure S1.** Moderator role of cardiorespiratory fitness and normalized handgrip strength in the association between sugar-sweetened beverage consumption and adiposity. A Moderation model for boys. B Moderation model for girls. Adjusted for sex, age, socioeconomic status, somatic maturity, fat-free mass, fruit consumption, vegetables consumption, and sweets consumption.
