# Supplementary figures and images for: The moderating role of physical fitness in the relationship between sugar-sweetened beverage consumption and adiposity in schoolchildren
Source: Sci Rep. 2022 Nov 3;12:18630. doi: 10.1038/s41598-022-23092-1 (PMC9633592; doi:10.1038/s41598-022-23092-1)

A

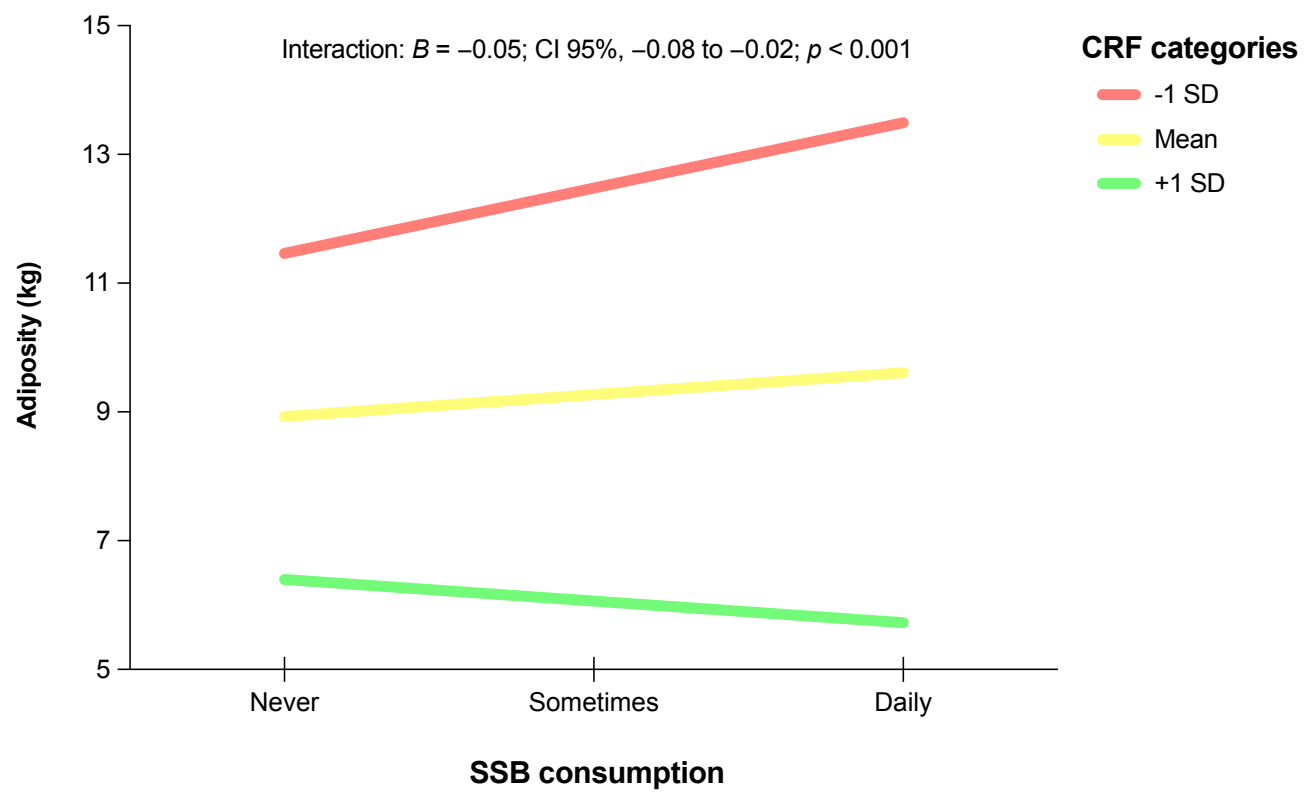

B

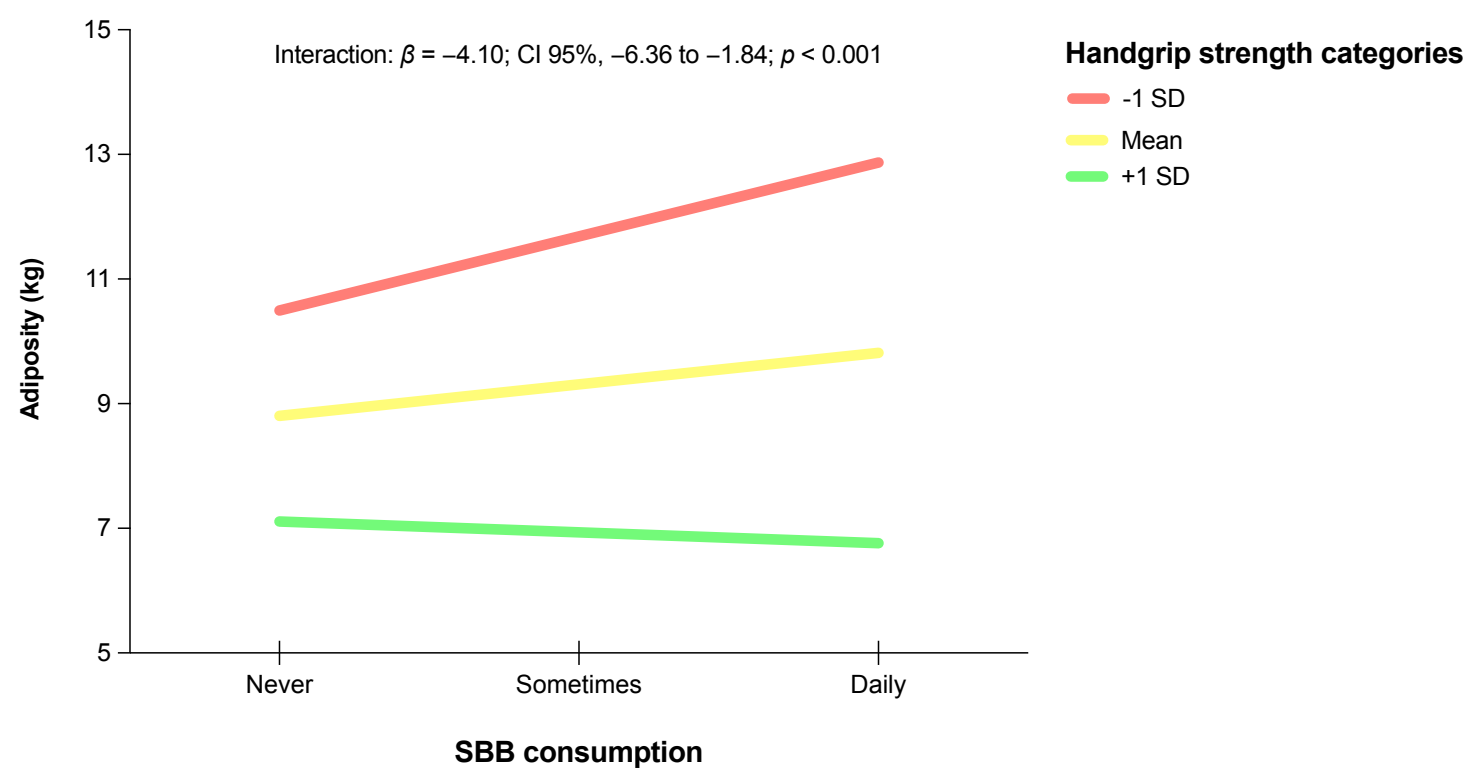

Supplement: Supplementary file 2 — Supplementary Figure S1. [file 41598_2022_23092_MOESM2_ESM.pdf]
